# Extramedullary Acute Myeloid Leukemia Presenting With Pericardial Effusion and Arrhythmias

**DOI:** 10.7759/cureus.39836

**Published:** 2023-06-01

**Authors:** Usman S Najam, Angela Khidhir, Leonard Palatnic, Farhan Azad

**Affiliations:** 1 Internal Medicine, University at Buffalo Jacobs School of Medicine and Biomedical Sciences, Buffalo, USA

**Keywords:** cardiac echo, onco-cardiology, cardiac tumor in adults, acute pericardial effusion, acute myeloid leukemia (aml), cardiac arrythmia

## Abstract

Acute myeloid leukemia (AML) is a hematologic malignancy that, through clonal transformation, results in abnormal proliferation and accumulation of immature myeloid cells in the bone marrow and blood. It is the most common type of acute leukemia in adults; however, extramedullary relapse is rare, and clinically significant metastasis to the heart with multiple presentations is even more infrequent. We present a case of a patient with AML, who, after successful treatment and remission, was found to have extramedullary metastasis in the form of one pericardial and two intracardiac masses, as well as a large pericardial effusion and conduction abnormalities.

## Introduction

Cardiac tumors are rarely encountered in clinical practice and pose a challenge in diagnoses and treatment. Of the distinct types of tumors that may occur, secondary tumors are reported to be about 20 times more common than primary tumors [1]. Although nearly all malignant cancers have the potential to metastasize to the heart, the most common are carcinoma (lung, breast, esophagus), melanoma, lymphomas, and lastly, leukemias [2]. Notably, lymphoproliferative disorders appear in 9.4% of cases with cardiac metastasis (including autopsies of affected patients), especially with myocardial (64%) and epicardial (44%) involvement [3]. With the likelihood of a cardiac tumor being secondary to metastasis, its presence likely indicates a dismal outlook due to the terminal stage that metastasis signifies [4]. These patients may go undiagnosed for many years prior to its discovery, and it is usually discovered after relapse of successful treatment, or in many cases, not discovered at all until autopsy. 

Acute myeloid leukemia (AML) is a hematologic malignancy that, through clonal transformation, results in the abnormal proliferation and accumulation of immature myeloid cells in the bone marrow and blood. It is the most common acute leukemia in adults; however, extramedullary relapse is considered rare, and clinically significant metastasis to the heart is even more infrequent [5]. We discuss a case of a patient with AML, who, after successful treatment and remission, was found to have extramedullary metastasis in the form of one pericardial and two intracardiac masses, as well as a large pericardial effusion on chest CT. The patient’s clinical course was then complicated by atrial fibrillation (AF) with a rapid ventricular response (RVR), which would intermittently convert to sinus bradycardia with long pauses when treated with rate-controlling medications.

## Case presentation

A 68-year-old male with a history of stage IV squamous cell carcinoma of the right tonsil status post radiation and chemotherapy with cisplatin presented to his primary care physician with worsening dyspnea and weakness. He was advised to report to a nearby hospital where his complete blood count was significant for an elevated level of immature white blood cells (WBC), with a WBC count of about 150,0000 with 37% blasts.

A bone marrow biopsy and aspirate were conducted after this finding, which revealed AML with 78% blasts. The patient was transferred to the local cancer center, where a repeat bone marrow biopsy and aspirate revealed secondary AML with 34% blasts, mixed-lineage leukemia gene-positive. Cytogenetics revealed 46 XY tetrasomy 8 in 12 cells, 46 pathologic XY in nine cells, 47 XY addition 11 in one cell, and 46 XY der10 in four cells examined. He was found to be FLT-3- and NPM-1-negative. Based on these findings, he received induction therapy with a 7+3 regimen that consisted of cytarabine for seven days, along with anthracycline for the initial three days. Day-14 bone marrow biopsy and aspirate revealed 78% blasts. The patient then went on to receive CLAG-M (cladribine, cytarabine, filgrastim, mitoxantrone) reinduction. Bone marrow biopsy and aspirate status post-reinduction with CLAG-M revealed AML in remission. As it had been four months since his initial diagnosis, the patient underwent a matched unrelated donor allogeneic peripheral blood stem cell transplant with a preparatory conditioning regimen of fludarabine and melphalan. He then underwent a bone marrow biopsy and aspirate about 400 days post-transplant, revealing less than 1% blasts, 100% donor, 500/500 female with flow cytometry being negative.

**Figure 1:**
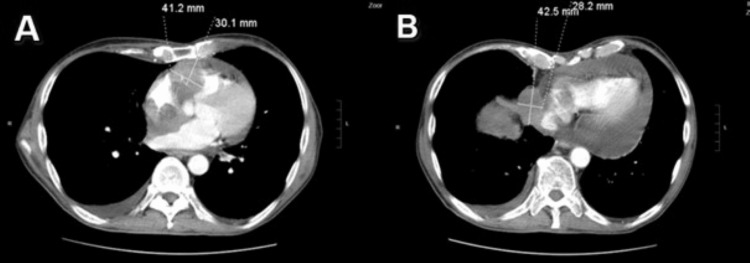
Chest CT (A) Showing 41.2 x 30.1-mm intracardiac mass causing indentation and marked mass effect on the superior and anterior aspect of the right ventricle. (B) Right pericardial mass measuring 4.2 x 2.8 cm abutting the right lateral aspect of the right ventricle and the junction of the SVC CT: computed tomography; SVC: superior vena cava

About two months after the patient's negative cytometry, he presented to his oncologist with new symptoms of weakness and shortness of breath with a physical exam significant for a sternal protuberance. He then underwent a CT scan, which revealed a pericardial mass and effusion along with two right cardiac masses. There was a significant mass effect on adjacent portions of the heart and severe narrowing of the inferior portion of the superior vena cava. Although one of these three masses had a right pericardiac location, the other two were thought to be intrinsic to the right atrium and right ventricle, causing a significant mass effect on these cardiac chambers and invading the corresponding portions of the right atrium and right ventricle (Figures 1A, 1B). The scan also showed marked enlargement of the pericardium with a large pericardial effusion, which was thought likely to be malignant. 

**Figure 2:**
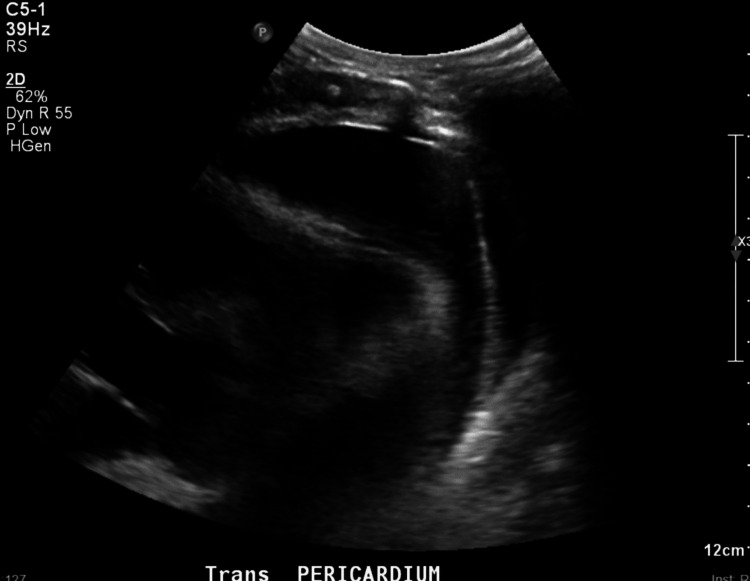
Transthoracic echocardiogram The image shows malignant pericardial effusion space prior to pericardiocentesis with the removal of 120 mL of fluid

The patient was subsequently admitted to the ICU where he underwent transthoracic echocardiogram (TTE)-guided pericardiocentesis with 120 mL of fluid removed (Figure 2). Cytology returned positive for numerous myeloblasts suggesting extramedullary relapse of his AML. The blasts were positive for myeloperoxidase with flow cytometry revealing an abnormal population expressing CD45(d), CD4(d), CD11c(h), CD33, CD38, CD11b(s), CD15, CD7(d), CD32, CD64(s), CD71, and CD163(s), and negative for HLA-DR, CD34, and CD52. Bone marrow biopsy at that time showed no evidence of disease.

He underwent further imaging with a positive emission tomography scan revealing hypermetabolic mediastinal lesions involving the great vessels, pericardium, and heart base. At this point, a "goals of care" discussion was held explaining the poor prognosis his metastasis portrayed. After expressing understanding, the patient decided that he would like to pursue a conservative treatment plan that favored a greater quality of life for the remaining time he had left. As the decision was made to forgo aggressive chemotherapy, the patient underwent palliative radiation with 24 Gy in 12 fractions to his chest and a 10-day course of decitabine, which was explained to be a less aggressive form of chemotherapy and better tolerated. 

Of note, the patient had interval admissions to the hospital for AF with RVR, which was initially treated with rate-controlling medications; however, he began to develop dangerously long pauses and, for this reason, it was decided not to treat the patient’s tachycardia. Despite being off rate-control agents, the patient continued to have paroxysmal sinus bradycardia and sinus arrest episodes, although asymptomatic. It was believed that the leukemic cells infiltrated the sinus node and the patient’s conduction system was causing his arrhythmia. Pacemaker placement was considered but was deemed to be challenging given the extent of the infiltration. Likewise, he was not a candidate for transvenous pacing due to the risk of tumor embolization. The patient was ultimately admitted for neutropenic fever and AF with RVR. His course was complicated by septic shock with pancytopenia secondary to severe bacteremia, bilateral non-loculated pleural effusions, and portal vein thrombosis. As the patient continued to decompensate, an extensive discussion was held with the family, and a decision was made to put him on comfort care. The patient ultimately passed away, about two months after the discovery of his cardiac metastasis.

## Discussion

Cardiac tumors, although mostly found to be secondary and labeled as benign, can have detrimental effects on patients. The initial diagnosis can be challenging due to non-specific symptoms and, for this reason, the condition may go overlooked or even undiagnosed [3]. In post-mortem studies, it has been reported that 30-44% of patients with leukemia may have cardiac involvement; however, about 99% of these are reported to be asymptomatic [6]. The initial presentation varies from case to case and may include hemodynamic compromise due to mass effect, a pericardial effusion on imaging, conduction disease with symptomatic electrocardiogram findings, or even mediastinal protuberance on the physical exam [5]. Once a diagnosis is confirmed through fluid cytology, bone marrow, or tissue biopsy, treatment of these cardiac tumors is directed at the primary tumor with chemotherapy or radiation. Less often, resection may be considered when intracavitary masses have led to significant hemodynamic compromise or when the primary tumor is controlled, and the patient's prognosis is good [7].

Our case illustrates an extremely rare presentation of relapsed AML, which metastasized to the heart and presented as a mediastinal protuberance, a pericardial effusion, and infiltration of the conduction system. And although our patient did not seek aggressive chemotherapy, he did still receive treatment for his cardiac tumors with both radiation and decitabine. Although a quick improvement was seen in the size of his masses on follow-up imaging, this alone was not enough to substantially prolong his life. Ultimately, the patient developed AF with RVR coupled with intermittent episodes of sinus bradycardia with long pauses, which proved too difficult to control. These findings may have been attributable to the intracardiac masses disrupting his conduction pathway, or the patient's treatment with radiation inadvertently having led to radiation-induced heart disease. In approximately 5% of patients, radiation treatment can result in conduction system abnormalities manifesting as atrioventricular block, sinus node syndromes, and supraventricular and ventricular arrhythmias [8]. In our case, the cytological analysis of the patient’s pericardial effusion revealing numerous myoblasts was sufficient evidence to suggest that the cardiac masses were malignant in nature. As such, rather than due to radiation, the arrhythmias were likely a consequence of extramedullary relapse.

In contrast to the patient's conduction abnormalities, his management highlighted the successful treatment of his malignant pericardial effusion. Malignant pericardial effusions are found in about 12-15% of patients with metastasis to the pericardium. The mechanism by which this happens is thought to be through direct extension or metastatic spread via lymphatics or blood, chemotherapeutic toxicity, radiation toxicity, or opportunistic infections secondary to immunosuppression [9]. The effects of these effusions can be as severe as hemodynamic compromise and cardiogenic shock, or as benign as dyspnea and discomfort. Management can be guided by the 2015 European Society of Cardiology guidelines, which recommend initially determining if there is hemodynamic instability with tamponade or if there is a suspected bacterial or neoplastic etiology. If either of these is the case, then pericardiocentesis for therapeutic or diagnostic purposes would be the appropriate step [10]. This was the strategy adopted in our patient, with resolution and minimal re-accumulation on follow-up imaging.

Our literature review revealed that it was exceedingly rare to see relapse with a solid cardiac mass in leukemia, with most cases occurring in lymphoblastic leukemia [11], particularly after hematopoietic stem-cell transplantation. Only a handful of cases showed relapse manifesting as a solid heart mass in AML, with Facenda-Lorenzo et al. describing the potentially devastating extent of relapse, as well as contributing clinical evidence to the understanding of cardiac metastasis [5]. The presentation consisted of a single large mass in the RA extending into the RV causing pericardial effusion and conduction abnormalities. Similar to the approach that was taken with our patient, a CT and TTE were used for imaging; however, in addition to this, a cardiac MRI was used for further detailing. To continue, rather than using cytology of the presenting pericardial effusion, their case involved an atrial biopsy to confirm the diagnosis. Regarding management, their patient also had conduction abnormalities in the form of slow AF, which was managed with isoproterenol, while the mass was managed with chemotherapy. Our case report, notably, expands on the severity of extramedullary relapse in AML by depicting a unique presentation of cardiac metastasis, providing clinicians an outlook on not only the consequences of leukemic infiltration of the heart but also the ensuing difficulty in its management. Simply put, irrespective of the differences between these two cases, the disease similarly led to the patient’s ultimate demise.

## Conclusions

To the best of our knowledge, this is the first case report highlighting extramedullary relapse of AML resulting in three cardiac masses with associated mass effect, a large pericardial effusion, and multiple conduction abnormalities such as difficult-to-rate-control AF and bradycardia with long pauses. And although clinically significant involvement of the myocardium in this population is rare, this case demonstrates how it is paramount to consider cardiac metastasis when cardiac symptoms do arise. Swift recognition and treatment may have a significant impact on a patient’s remaining quality of life. And as more cases are reported and further research is conducted, we should expect to see a greater length of survival and improved quality of life for these patients in the future.

## References

[1] Lam KY, Dickens P, Chan AC (1993). Tumors of the heart. A 20-year experience with a review of 12,485 consecutive autopsies. Arch Pathol Lab Med.

[2] Burazor I, Aviel-Ronen S, Imazio M (2018). Metastatic cardiac tumors: from clinical presentation through diagnosis to treatment. BMC Cancer.

[3] Bussani R, De-Giorgio F, Abbate A, Silvestri F (2007). Cardiac metastases. J Clin Pathol.

[4] Welch DR, Hurst DR (2019). Defining the hallmarks of metastasis. Cancer Res.

[5] Facenda-Lorenzo M, Sánchez-Quintana A, Quijada-Fumero A (2016). Cardiac relapse of acute myeloid leukemia after allogeneic hematopoietic stem cell transplantation. Case Rep Oncol Med.

[6] Luo Z, Cheng J, Wang Y (2022). Cardiac infiltration as the first manifestation of acute lymphoblastic leukemia: a systematic review. Front Oncol.

[7] Tyebally S, Chen D, Bhattacharyya S (2020). Cardiac tumors: JACC CardioOncology state-of-the-art review. JACC CardioOncol.

[8] Wang H, Wei J, Zheng Q, Meng L, Xin Y, Yin X, Jiang X (2019). Radiation-induced heart disease: a review of classification, mechanism and prevention. Int J Biol Sci.

[9] Refaat MM, Katz WE (2011). Neoplastic pericardial effusion. Clin Cardiol.

[10] Lazaros G, Vlachopoulos C, Lazarou E, Tsioufis K (2021). New approaches to management of pericardial effusions. Curr Cardiol Rep.

[11] De Lazzari M, Fedrigo M, Perazzolo Marra M (2015). Relapsing leukemia infiltrating the heart. Circ Heart Fail.

